# Octahedral Shaped PbTiO_3_-TiO_2_ Nanocomposites for High-Efficiency Photocatalytic Hydrogen Production

**DOI:** 10.3390/nano11092295

**Published:** 2021-09-03

**Authors:** Simin Yin, Shun Liu, Yongfeng Yuan, Shaoyi Guo, Zhaohui Ren

**Affiliations:** 1 School of Mechanical Engineering and Automation, Zhejiang Sci-Tech University, Hangzhou 310018, China; ls19980104@163.com (S.L.); yuanyf@zstu.edu.cn (Y.Y.); syiguo@163.com (S.G.); 2 State Key Lab of Silicon Materials, School of Materials Science and Engineering, Zhejiang University, Hangzhou 310027, China; renzh@zju.edu.cn

**Keywords:** hydrothermal, anatase TiO_2_, ferroelectric, octahedron, H_2_ production

## Abstract

In this work, octahedral shaped PbTiO_3_-TiO_2_ nanocomposites have been synthesized by a facile hydrothermal method, where perovskite ferroelectric PbTiO_3_ nanooctahedra were employed as substrate. The microstructures of the composites were investigated systemically by using XRD, SEM, TEM and UV-Vis spectroscopy. It was revealed that anantase TiO_2_ nanocrystals with a size of about 5 nm are dispersed on the surface of the {111} facets of the nanooctahedron crystals. Photocatalytic hydrogen production of the nanocomposites has been evaluated in a methanol alcohol-water solution under UV light enhanced irradiation. The H_2_ evolution rate of the nanocomposites increased with an increased loading of TiO_2_ on the nanooctahedra. The highest H_2_ evolution rate was 630.51 μmol/h with the highest concentration of TiO_2_ prepared with 2 mL tetrabutyl titanate, which was about 36 times higher than that of the octahedron substrate. The enhanced photocatalytic reactivity of the nanocomposites is possibly ascribed to the UV light absorption of the nanooctahedral substrates, efficient separation of photo-generated carriers via the interface and the reaction on the surface of the TiO_2_ nanocrystals.

## 1. Introduction

Photocatalytic splitting of water into H_2_ by using semiconductor materials is a promising and alternative method for clear energy generation [1,2,3,4]. Since 1972, TiO_2_ has been extensively explored as a potential photocatalytic semiconductor in the splitting of water, where great and consecutive efforts have been devoted into the improved reactivity for water splitting by catalytic design [5,6,7,8,9,10,11,12]. Despite great efforts, the efficiency for water splitting to generate H_2_ remains low at this stage due to the high recombination rate of photo-generated charge carriers in catalysts and the presence of oxidized and reduced intermediates in the reaction mixtures [13,14,15,16]. This low efficiency has been proved to be significantly limiting the applications of TiO_2_ in energy harvesting. To improve the H_2_ production efficiency by water splitting, various approaches have been developed to modify TiO_2_, such as the deposition of noble metal (Pt, Au, Pd), element doping and the surface sensitization by organic dyes. Particularly, compositing TiO_2_ with other semiconductors with a desirable band structure is highly attractive for improving the carrier separation [17,18,19,20,21,22,23,24,25].

Perovskite ferroelectric materials, characterized by a switchable spontaneous polarization, can provide a fascinating surface chemical environment to drive the growth of semiconductor nanostructures [26,27]. More interestingly, a c internal electric field in single-domain or polarized perovskites could be essential for enhancing separation of photo-generated carriers of the semiconductor catalyst [7,14,28]. Integrating perovskite substrates with the photocatalytic activity of the TiO_2_ makes it possible to increase the photocatalytic efficiency, including water splitting to generate H_2_. Accordingly, perovskite/titania composites have been the focus of many investigations, for instance, epitaxial growth of TiO_2_ on single-domain PbTiO_3_ nanoplates for H_2_ production [2], heterostructured PbTiO_3_-TiO_2_ core-shell particles for enhanced H_2_ evolution [29] and TiO_2_/BaTiO_3_ in the splitting of water [30]. In addition to these nanoparticles and nanoplates, perovskite PbTiO_3_ octahedrons with a size of 50–100 nm have been reported in our previous work with {111} exposed, leading to the unique visible light photocatalytic reactivity [31]. Motivated by the above advances in photocatalysts, we expect that these faceted nanooctahedra would be desirable substrates able to adjust the crystal growth of TiO_2_ and then fabricate composites for photocatalytic explorations.

In this work, we report for the first time the facile hydrothermal synthesis of octahedral shaped PbTiO_3_-TiO_2_ nanocomposites by employing perovskite PbTiO_3_ nanooctahedral crystals as substrates. It was revealed that the surface of the perovskite substrate crystals was covered by the as-grown TiO_2_ nanocrystals on {001} facets, adopting an anatase structure. The resulting PbTiO_3_-TiO_2_ nanocomposites displayed an enhanced photocatalytic performance in splitting of water to generate H_2_, with the highest evolution rate of 630.51 μmol/h. On the basis of these results, the PbTiO_3_ substrates are expected to be crucial for the enhanced photocatalytic activity by an improved carrier separation and transportation to the active TiO_2_ nanocrystals due to an interfacial band bending. This work may provide the opportunity to the design of novel high efficient ferroelectric-based catalysts.

## 2. Materials and Methods

### 2.1. Synthesis

Firstly, octahedral shaped perovskite PbTiO_3_ crystals were synthesized by a modified Li^+^-assisted hydrothermal reaction [31,32]. Then PbTiO_3_-TiO_2_ nanocomposites were prepared by using the perovskite PbTiO_3_ nanooctahedral crystals as substrates and tetrabutyl titanate (TBOT) as Ti^4+^ source via a hydrolysis-hydrothermal method. Briefly, precursors were prepared by mixing different volumes of TBOT (0, 0.5, 1.5 and 2.0 mL) with 25 mL absolute ethanol and strong stirring for 30 min. Then, 1.0 g hydrothermally synthesized perovskite PbTiO_3_ nanooctahedral crystals were added into the obtained solutions for another 120 min stirring to obtain homogeneous suspensions. NH_3_·H_2_O was introduced as mineralizer and the whole volume of the suspension was adjusted to 35 mL by adding deionized water. Thereafter, the suspensions were transferred to 50 mL Teflon-lined autoclaves and maintained at 200 °C for 12 h. After natural cooling to room temperature, the resulting samples were collected, washed with ethanol and deionized water respectively for several times, and then dried at 60 °C for 12 h. The samples prepared with different TBOT were denoted as S1(TBOT: 0.5 mL), S2(TBOT: 1.5 mL), S3(TBOT: 2.0 mL), respectively, ready for characterization.

Pt-loaded samples for photocatalytic H_2_ generation were prepared by a chemical reduction method. Typically, the as-prepared PbTiO_3_-TiO_2_ nanocomposites were dispersed in deionized water under strong sonication to form a slurry mixture. Then, an aqueous solution of H_2_PtCl_6_·6H_2_O (1 wt% of Pt) was added dropwise to the above PbTiO_3_-TiO_2_ slurry, and after a 15-min ultrasonic bath, an aqueous NaBH_4_ solution was slowly added. The resulting solution was kept in the ultrasonic bath for another 15 min, washed and filtered, and finally dried at 60 °C for 12 h.

### 2.2. Characterization

The as-synthesized samples were systematically investigated and characterized by X-ray diffraction (XRD, ARLXTRA, Thermo, Olten, Switzerland, CuK_α_), field emission scanning electron microscope (field emission SEM, S-4800, Hitachi, Tokyo, Japan) and TEM (F20 using an accelerating voltage of 200 kV, FEI, Portland, OR, America). Thermogravimetry (TG) and differential scanning calorimetry (DSC) analysis was carried out on a TA-SDT (Q600 V8.2 Build 100) instrument (TA Instruments, New Castle, DE, USA). The UV-Vis diffuse reflectance spectra were recorded using a UV-3600 UV-VIS-NIR spectrophotometer (Shimadzu, Kyoto, Japan).

### 2.3. Photocatalytic H_2_ Generation

The photocatalytic H_2_ evolution from a methanol aqueous solution was conducted in a 100 mL quartz tube. The photocatalyst powders (30 mg) were dispersed in a methanol/deionized water solution (20 mL:80 mL) in a quartz tube under stirring. The solution was then purged with N_2_ for at least 30 min to remove O_2_ and then sealed with a rubber septum. The light source was a 500 W high-pressure mercury lamp (XPA-7) photochemical reactor (Nanjing Xujiang Machine-electronic Plant, Nanjing, China), and the average UV light intensity was ca. 45 mW/cm^2^. The temperature of the suspension during irradiation was maintained at 25 °C using a thermostatically controlled water bath. The amount of H_2_ was determined using a Shimadzu GC-2014 gas chromatography system (N_2_ carrier gas, molecular sieve 5 Å, TCD detector).

## 3. Results and Discussion

**Figure 1:**
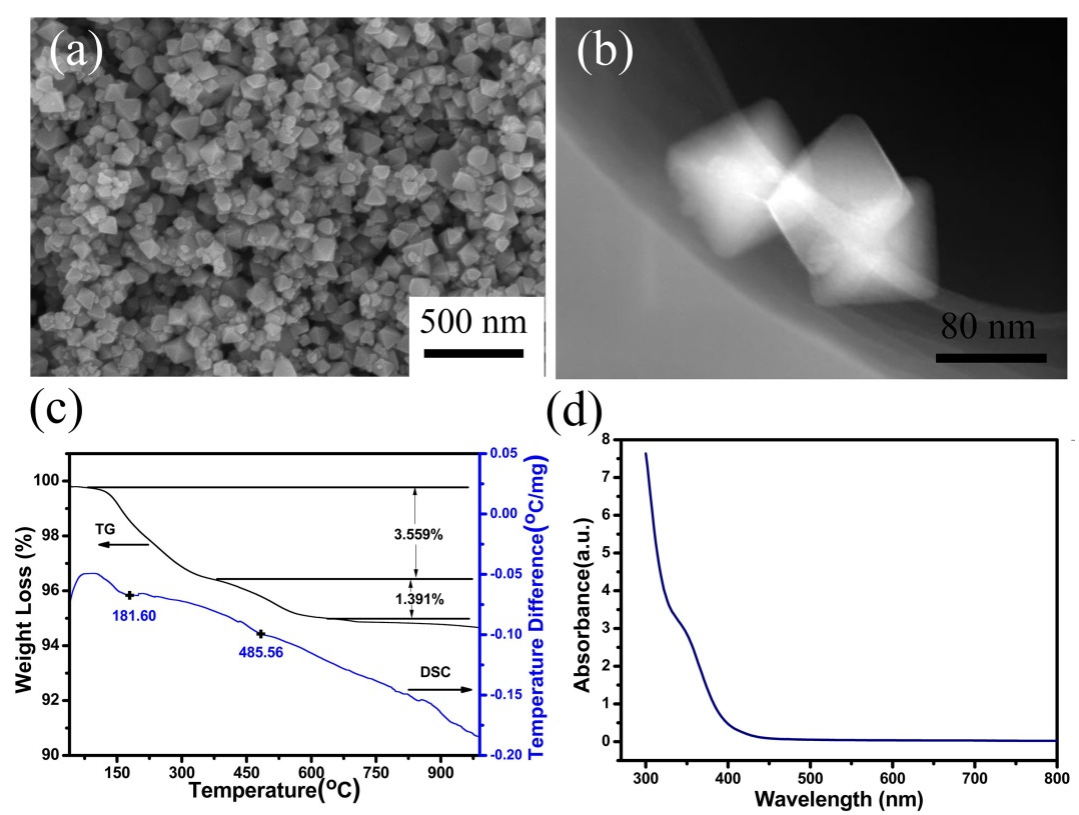
(**a**) SEM and (**b**) HAADF-STEM images, (**c**) TG-DSC curves and (**d**) UV-Vis absorption spectrum of the hydrothermally synthesized perovskite PbTiO_3_ nanooctahedron crystals.

Figure 1a,b present the SEM and HAADF-STEM images of hydrothermally synthesized PbTiO_3_ nanooctahedral crystals, respectively. SEM image indicates that the sample consists of large-scale nanocrystals with smooth surface, sharp edges and regular facets exposed. HAADF-STEM image shows the magnified projections of three PbTiO_3_ nanocrystals from different orientations. From these results, it can be found that the nanocrystals all adopt an octahedral shape, with a size about 50–100 nm.

Figure 1c presents the TG-DSC curves of the as-synthesized PbTiO_3_ nanooctahedral crystals. Two peaks can be observed from the DSC curve. The first peak located at 181.6 °C can be assigned to physically absorbed water evaporation or the decomposition of intermediate products [33]. The peak at about 485.56 °C was determined to be the Curie temperature of the PbTiO_3_ nanooctahedra, where a phase transition process from a ferroelectric tetragonal phase to paraelectric cubic one occurred. This Curie temperature is very close to the reported value of the counterpart bulk PbTiO_3_ [34], suggesting the ferroelectric property of the as-prepared PbTiO_3_ nanooctahedra. Figure 1d displays the UV-Vis spectrum of the as-prepared PbTiO_3_ nanooctahedra, the energy band gap is calculated to be 2.65 eV, matching well with the reported value [32].

**Figure 2:**
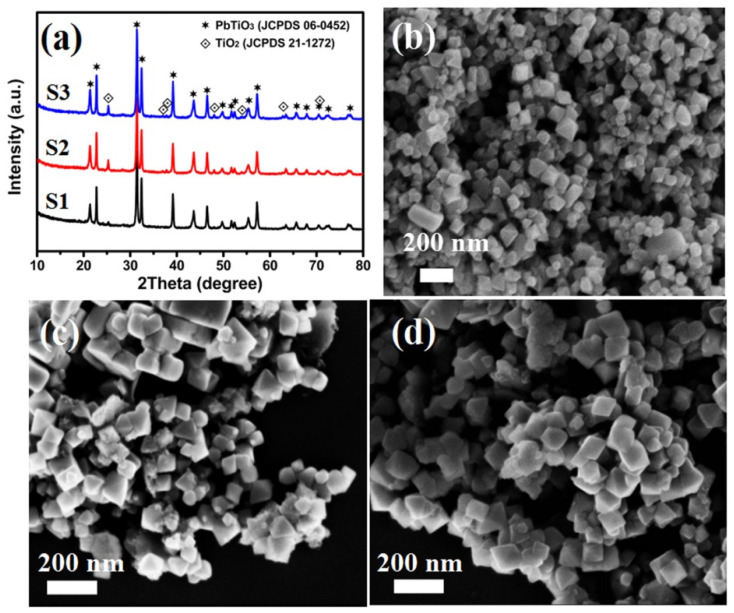
(**a**) XRD patterns of as-prepared PbTiO_3_-TiO_2_ nanocomposite samples: S1, S2 and S3; (**b**–**d**) SEM images of as-prepared samples: S1, S2 and S3. (S1: TBOT: 0.5 mL, S2: TBOT: 1.5 mL, S3: TBOT: 2.0 mL).

X-ray diffraction patterns of the as-prepared nanocomposite samples were collected and are shown in Figure 2a. All the diffraction peaks can be well indexed to the standard patterns of PbTiO_3_ (JCPDS: 06-0452) and anatase TiO_2_ (JCPDS: 21-1272), respectively, indicating a two-phase composite. The strong diffraction peaks argue a good crystallinity of the samples, and no diffraction peaks of other impurities could be observed. One should note that the diffraction peak intensity of anatase TiO_2_ (101) near 2θ = 25.28° in sample S1, S2 and S3 gradually increases, indicating an increase content of anatase TiO_2_ in the nanocomposite samples due to the increase of the starting reagent of TBOT.

Figure 2b–d present the corresponding SEM images of the as-prepared nanocomposites S1, S2 and S3. It can be observed that all the samples exhibited faceted octahedral configurations with particle sizes in the range of 50–100 nm. The surface of the octahedra was covered by a layer of homogenously dispersed nanoparticles. The sharp edge of the as-synthesized PbTiO_3_ octahedron crystals changes to be curved with the compositing of TiO_2_ on the surface. At this stage, free-standing nanoparticles are difficult to be observed from SEM image. This fact suggests that anatase TiO_2_ determined from XRD in Figure 2a was already integrated with the perovskite PbTiO_3_ octahedrons to form a PbTiO_3_-TiO_2_ nanocomposite. From the combined results from XRD and SEM, it can be confirmed that the as-prepared samples are octahedral shaped PbTiO_3_-TiO_2_ nanocomposites.

**Figure 3:**
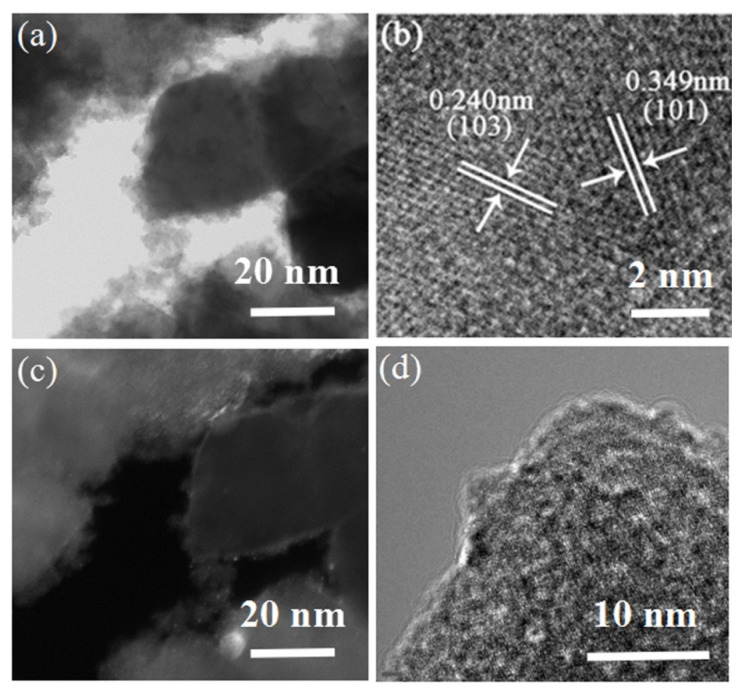
(**a**–**d**) TEM and HRTEM image of PbTiO_3_-TiO_2_ nanocomposite by TBOT: 2. 0 mL (S3).

To further investigate the detailed microstructure of the PbTiO_3_-TiO_2_ nanocomposites, TEM and HRTEM images were analyzed. Figure 3a,c show the low-magnification TEM images of specific octahedral shaped PbTiO_3_-TiO_2_ nanocomposites (S3) by a bright field mode and dark field mode, respectively. It can be observed that the octahedral substrate presents a specific parallelogram projection, with a continuous and flurry layer grown on the surface, surrounding the parallelogram projection. The anatase TiO_2_ (JCPDS: 21-1272) nanocrystals with a size of about 5 nm are attached to the faceted surfaces of the substrates. Figure 3b,d present HRTEM images of the nanocomposite. The lattice spacing of 0.240 nm and 0.349 nm, denoted in Figure 3b, can be indexed to the anatase planes of (103) and (101), respectively. Hence, it can be convinced that the anatase TiO_2_ nanocrystals grew on the surface of the perovskite octahedron crystals to form an octahedral shaped PbTiO_3_-TiO_2_ nanocomposite.

**Figure 4:**
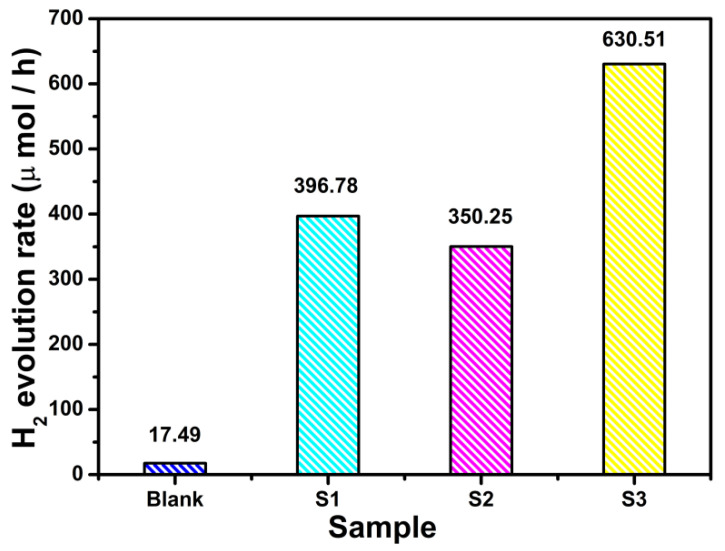
The photocatalytic H_2_ evolution rate of water splitting under UV light (λ < 420 nm) irradiation of as-prepared samples: blank sample, S1, S2 and S3.

The photocatalytic activity of the as-prepared octahedrally-shaped PbTiO_3_-TiO_2_ nanocomposites was evaluated by the H_2_ evolution reaction of water splitting under UV light (λ < 420 nm) in 2 h, where methanol alcohol was used as sacrificial reagent and 30 mg photocatalyst powders were employed each time. As shown in Figure 4, the H_2_ evolution rate for pristine PbTiO_3_ nanooctahedra at 2 h was only 17.49 μmol/h, indicating a relatively low photocatalytic activity in water splitting. As a comparison, the PbTiO_3_-TiO_2_ nanocomposites (S1: TBOT 0.5 mL, S2: TBOT 1.5 mL, S3: TBOT 2.0 mL) exhibited a remarkable photocatalytic reactivity in H_2_ evolution, where significant H_2_ bubbles have been observed during the water splitting reaction process. The photocatalytic reactivity of H_2_ evolution was greatly enhanced with the increasing use of TBOT. In particular, S3 exhibited the highest H_2_ generation rate of 630.51 μmol/h (at 2 h), which is approximately 36 times higher than that of the blank sample within 2 h.

**Figure 5:**
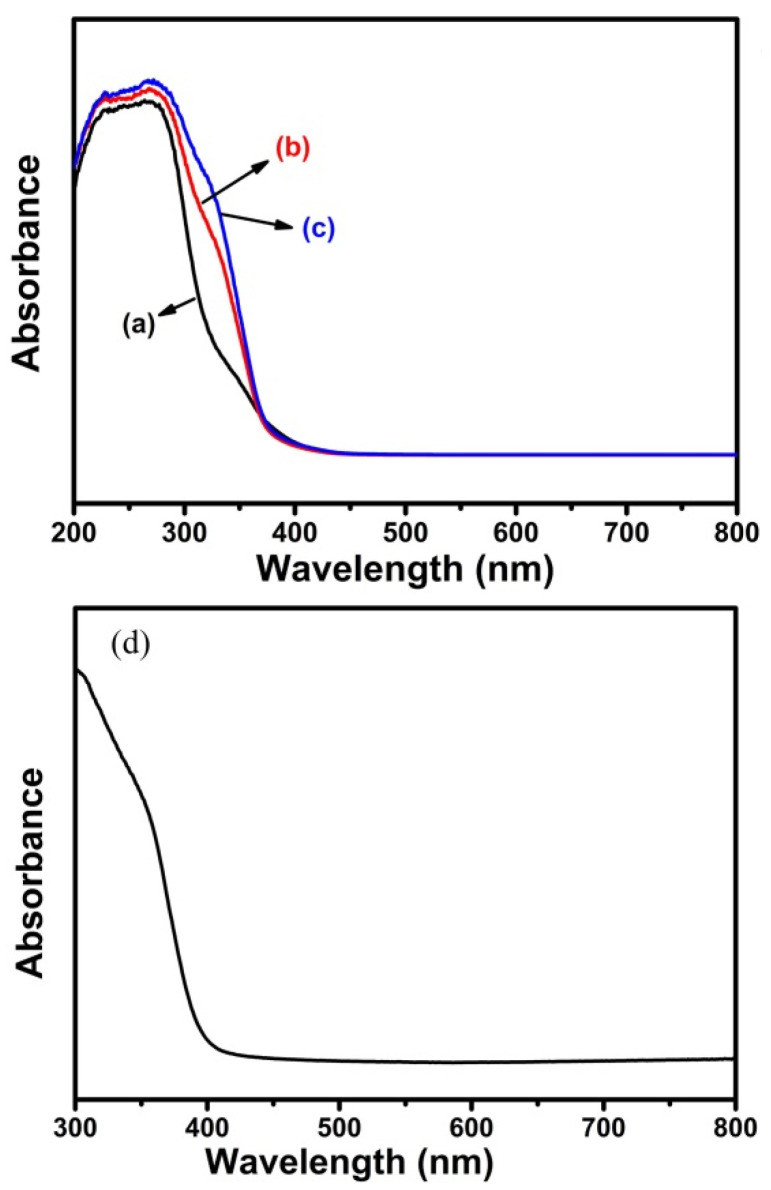
UV-Vis absorption spectra of the as-synthesized sample of (**a**) S1, (**b**) S2, (**c**) S3 and (**d**) TiO_2_ synthesized with TBOT via hydrothermal method as a control experiment.

The enhancement of H_2_ generation rate could be originated from the interfacial band structure of the nanocomposites. Thus, the UV-Vis absorption spectra in Figure 5 were analyzed to further investigate the optical property of the nanocomposites and pristine TiO_2_. Figure 5a–c show the UV-Vis absorption spectra of the PbTiO_3_-TiO_2_ nanocomposite samples. It can be observed that all the nanocomposite samples S1, S2 and S3 have similar onset absorption which varied slightly, and they all exhibited little absorbance of light with wavelength longer than 400 nm. However, the amount of light absorption in the section of 300 nm–400 nm wavelength by S1, S2 and S3 gradually increased, which may be assigned to the increased amount of perovskite substrate-anatase TiO_2_ interfaces in the nanocomposite samples.

This increased absorption could also lead to a higher efficiency in photogeneration of charge carriers. The absorption band gap of the sample S3 was estimated to be 3.16 eV.

As shown in Figure 5d, the UV-Vis spectrum of the TiO_2_ sample synthesized with TBOT was also provided as a comparison. It could be observed that the hydrothermally synthesized TiO_2_ exhibited very small absorbance in the range of 400 nm–800 nm and the onset of the absorption is approximately near 393 nm, where the band gap was determined to be about 3.15 eV, matching well with the reported value previously. Compared with the pure anatase sample, the absorption of the nanocomposite samples increased in the order of S1 < S2 < S3. Combined with the absorption band edge of pure PbTiO_3_ nanooctahedra (Figure 1d), it can be deduced that the PbTiO_3_ substrate could affect the light absorbancy and charge carrier generation. Moreover, with the introduction of the substrate in the PbTiO_3_-TiO_2_ nanocomposites, the intrinsic spontaneous polarization could possibly adjust the PbTiO_3_-titania interfaces and the band bending, facilitating the carriers transferring from the PbTiO_3_ substrate to anatase TiO_2_ [35].

**Figure 6:**
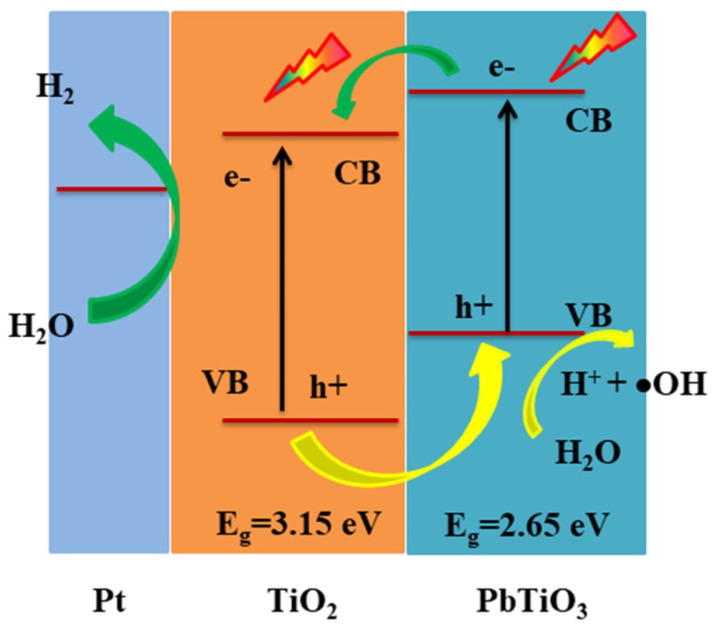
Proposed mechanism for the photocatalytic H_2_ evolution in water splitting over octahedral shaped PbTiO_3_-TiO_2_ nanocomposites.

On the basis of the above analysis, a possible photocatalytic mechanism of PbTiO_3_-TiO_2_ nanocomposites was proposed and schematically presented in Figure 6. Under UV light irradiation, the light absorption occurred spontaneously in both of the PbTiO_3_ nanooctahedra and TiO_2_ nanocrystals in the PbTiO_3_-TiO_2_ nanocomposites. Then the photo-generated electrons and holes transferred to TiO_2_ and PbTiO_3_, respectively. Specifically, the crystalline anatase TiO_2_ grown on the {111} facets of the octahedron was excited and the photo-generated electrons and holes were separated by an interfacial band bending due to the existence of spontaneous polarization of the perovskite support [35]. In addition, the PbTiO_3_ substrate can also be excited to generate photo-generated carriers which could further be transferred to the surface of TiO_2_ via the interface and contributed to H_2_ generation. Thus, the increased content of the photogenerated carriers in the PbTiO_3_-TiO_2_ nanocomposites and the decreased recombination of electrons and holes could synergistically improve the photocatalytic activity of the nanocomposites.

## 4. Conclusions

In conclusion, octahedrally shaped PbTiO_3_-TiO_2_ nanocomposites have been successfully synthesized and the photocatalytic hydrogen production in splitting of water was explored. The as-prepared nanocomposites exhibit a remarkable photocatalytic H_2_ generation activity in splitting of water and the highest H_2_ evolution rate was about 630.51 μmol/h, which is much higher that of pristine samples. A possible mechanism based on the band structure of the composite interface was proposed. The enhanced photocatalytic reactivity could be attributed to the absorption of the UV light (λ < 420 nm) by the perovskite PbTiO_3_ substrates, the separation of photo-generated carriers at the interface and reactions at the surface of the anatase TiO_2_ nanocrystals.

## Data Availability

The data presented in this study are available on request from the corresponding author. The data are not publicly available due to the reason that the data also forms part of an ongoing study.
